# Computational modeling of spinal neural circuits involved in transition to hopping pattern in EphA4 knock-out mice

**DOI:** 10.1186/1471-2202-14-S1-P339

**Published:** 2013-07-08

**Authors:** Natalia A Shevtsova, Ole Kiehn, Ilya A Rybak

**Affiliations:** 1 Department of Neurobiology and Anatomy, Drexel University College of Medicine, Philadelphia, PA, USA; 2 Department of Neuroscience, Karolinska Institute, Stockholm, Sweden

## 

**Figure 1:**
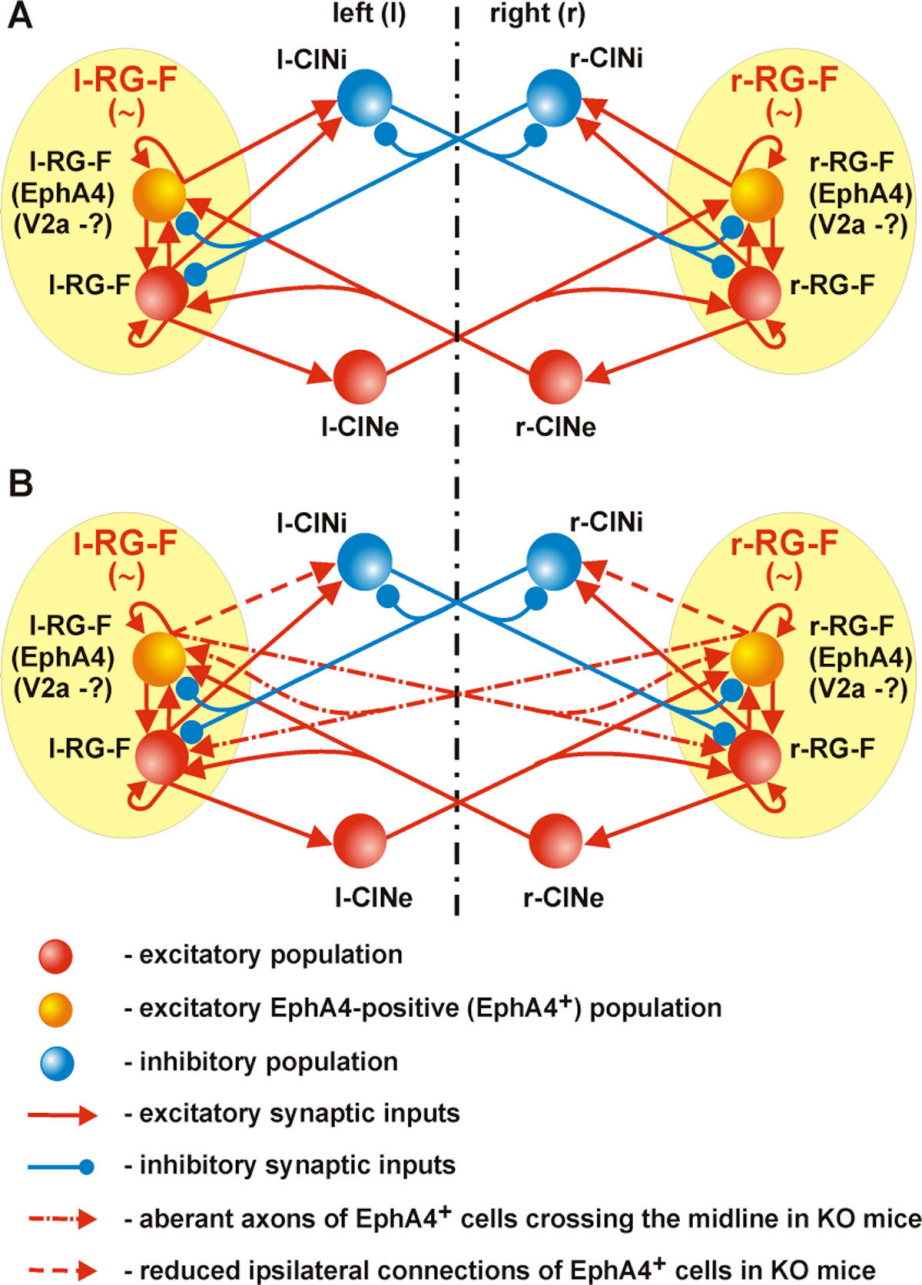
Schematic of spinal circuits in the model for a normal (A) and EphA4 KO (B)

According to resent studies [1,3] the spinal cord neural circuits involved in left-right coordination of locomotor activity include (a) excitatory and inhibitory commissural interneurons (CINs) with axons crossing the midline and (b) excitatory (glutamatergic) interneurons with ipsilateral axon projections activating CIN pathways. The distribution and guidance of axonal projections of the glumatergic neurons during development critically depend on the EphA4 receptors, so that in EphA4 knock-out (KO) mice the normal left-right alternating pattern of walking is replaced with a hopping gait [3]. Isolated spinal cord preparations from EphA4 KO mice also exhibit a synchronized left and right locomotor activity [3]. The pharmacological enforcement of inhibition recovers the normal left-right alternating locomotor pattern in these preparations [3]. It was suggested [2] that switching to a left-right synchronized hoping pattern in the EphA4 KO mice might result from an abnormal midline crossing of axons of glutamatergic neurons that are normally distributed ipsilaterally. This suggestion has been directly confirmed in anatomical studies [4]. To further evaluate this idea we developed a computational model of neural circuitries in the spinal cord with left and right rhythm generators (RGs) containing sub-populations of EphA4-positive excitatory neurons projecting to the populations of CINs responsible for left-right alternation (see Figure 1). The model includes interacting populations of spinal interneurons modeled in the Hodgkin-Huxley style. To simulate changes in the EphA4 KO spinal circuits, the axons of the EphA4-positive neurons were redistributed and partly redirected to the contralateral side. As a result, the excitatory interactions between left and right RGs overcame their inhibitory interactions hence synchronizing their activity resulting in a hopping-like output pattern. A subsequent increase of inhibition in the model could recover the normal left-right alternating pattern. The model proposes a mechanistic explanation for the hopping gait in EphA4 KO mice and provides insights into the organization of the locomotor central pattern generator.
